# Investigation of the long-term sustainability of changes in appetite after weight loss

**DOI:** 10.1038/s41366-018-0119-9

**Published:** 2018-06-21

**Authors:** S Nymo, SR Coutinho, PH Eknes, I Vestbostad, JF Rehfeld, H Truby, B Kulseng, C Martins

**Affiliations:** 1 Obesity Research Group, Department of Cancer Research and Molecular Medicine, Faculty of Medicine, Norwegian University of Science and Technology (NTNU), Trondheim, Norway; 2 Centre for Obesity and Innovation (ObeCe), Clinic of Surgery, St. Olav University Hospital, Trondheim, Norway; 3 Department of Clinical Biochemistry, Rigshospitalet, University of Copenhagen, Copenhagen, Denmark; 4 Department of Nutrition, Dietetics & Food, Monash University, Melbourne, Australia

## Abstract

**Background/Objective:**

Diet-induced weight loss (WL) leads to a compensatory increase in appetite and changes in the plasma concentration of appetite-regulating hormones are likely to play a role. Whether these changes are transient or sustained remains unclear. This study aimed to assess if changes in subjective and objective appetite markers observed with WL are sustained after 1 year (1Y).

**Subjects/Methods:**

In total 100 (45 males) individuals with obesity (BMI: 37 ± 4 kg/m^2^, age: 43 ± 10 years) underwent 8 weeks (wks) of a very-low energy diet (VLED), followed by 4 wks refeeding, and a 1Y maintenance program. Fasting/postprandial subjective ratings of hunger, fullness, desire to eat, and prospective food consumption (PFC) were assessed, and plasma concentration of active ghrelin (AG), total peptide YY (PYY), active glucagon-like peptide 1, cholecystokinin (CCK), and insulin measured, at baseline, week 13 (Wk13) and 1Y.

**Results:**

At Wk13, 16% WL (−18 ± 1 kg, *P* < 0.001) was associated with a significant increase in fasting and postprandial hunger ratings (*P* < 0.01 and *P* < 0.05, respectively), and postprandial fullness (*P* < 0.01) combined with a reduction in PFC (*P* < 0.001). These were accompanied by a significant rise in basal and postprandial AG concentrations (*P* < 0.001, for both), a reduction in postprandial CCK (*P* < 0.01) and in basal and postprandial insulin (*P* < 0.001). At 1Y follow-up, with sustained WL (15%; −16 ± 1 kg, *P* < 0.001), fasting hunger and postprandial fullness ratings remained increased (*P* < 0.05 for both), and postprandial PFC reduced (*P* < 0.001). Basal and postprandial AG remained elevated and insulin reduced (*P* < 0.001, for all), while postprandial CCK was increased (*P* < 0.01) and PYY decreased (*P* < 0.001).

**Conclusion:**

With a 15% sustained WL at 1Y, the drive to eat in the fasting state is increased, but this may be balanced out by raised postprandial feelings of fullness. To assist with WL maintenance, new strategies are required to manage increased hunger and drive to eat.

## Introduction

Worldwide >40% of all adults have attempted to control their body weight by weight loss strategies [1]. Weight loss (WL) maintenance is now the most challenging part within obesity management as relapse is common and only 10–20% succeed in maintaining their lower body weight long-term [2, 3]. Reasons for recidivism are poorly understood and likely to be complex, involving a combination of reduced motivation and compliance to restrict energy intake by dieting and increase energy expenditure via exercise regimens [4–7], together with metabolic, neuroendocrine and autonomic adaptive responses that oppose the reduced obese state [8–11].

The landmark papers by Leibel et al. [10] and Dulloo et al. [11] showed that WL is followed by a reduction in total energy expenditure (TEE) larger than predicted and, despite that, an increase in hunger and hyperphagia. Since then, reviews by Cornier et al. [8], Rosenbaum et al. [9] and Doucet et al. [12], among others, have described the compensatory mechanisms activated with WL, on both sides of the energy balance equation, which may contribute to weight re-gain.

A recent study has estimated that the increased appetite seen with WL is probably threefold larger than the corresponding reduction in TEE and likely the main driver of weight re-gain [13]. Several studies, that use visual analogue scales (VAS) to measure appetite, demonstrate that diet-induced WL, outside of ketosis, is associated with increased hunger feelings in the fasted state [14–16], likely mediated via an increase in plasma ghrelin concentration [15–17], even though some studies report no change [18, 19]. The impact of WL on fullness feelings [15, 16, 20] and secretion of peptides signalling satiety is, unfortunately, less clear [15, 16, 20, 21].

Whether the changes in appetite associated with WL are sustained in the long-term remains a point of debate, as the available research is inconsistent in its findings [15, 20, 21], with different methods to measure appetite being employed and different hormonal fractions being measured. Sumithran et al. [15] reported a sustained increase in subjects feelings of hunger using VAS, plus an increase in ghrelin plasma concentration at 1 year follow-up, as well as no change in postprandial concentration of active glucagon-like peptide 1 (GLP-1) and a reduction in total peptide YY (PYY), after an initial 12% WL followed by 50% regain. However, another study reported a sustained increase in both total GLP-1 and PYY_3–36_ postprandial secretion after a 12% sustained WL at 1 year follow-up [21], while another showed no change in active GLP-1 postprandial secretion after 6 months of sustained WL maintenance [20]. Moreover, from our knowledge, no study has determined if sex modulates the changes in appetite seen with WL. This is of great importance given that sex has been shown to modulate appetite sensations and the secretion of several appetite-related hormones [22, 23]. The primary aim of this study was to determine if changes in appetite (both subjective feelings of appetite using VAS and objective plasma concentrations of appetite-related hormones) seen after WL, are sustained at 1-year (1Y) follow-up. A secondary aim was to determine if sex modulates any changes in appetite.

## Methods

### Participants

Healthy adults with obesity (30 < BMI <50 kg/m^2^) from the local community of Trondheim, Norway, were recruited for this study by social media and articles in the local newspaper. The study was approved by the regional ethics committee (Ref., 2012/1901), registered in ClinicalTrial.gov (NCT01834859) and conducted according to the Declaration of Helsinki. All participants signed informed consent before participation.

Participants had to be weight stable (<2 kg change over the last 3 months), not dieting to lose weight and with a sedentary lifestyle. Exclusion criteria were pregnancy, breast-feeding, clinical significant illness, including diabetes, previous WL surgery and/medication known to affect appetite/metabolism or induce WL.

### Study design

This was a longitudinal intervention study with repeated measurements. Participants underwent an 8-week supervised very-low energy diet (VLED), followed by a 4-week refeeding phase, and a 1-year weight maintenance program (study diagram, Supplementary Figure I).

#### Weight loss phase

Participants followed for 8 weeks a VLED (Allévo, Karo Pharma AS, Sweden) with 550/660 kcal/day, for females and males respectively (carbohydrate 42%, protein 36%, fat 18% and fibre 4%), plus no-energy fluids and low starch vegetables (max 100 g/day) [16].

At week 9, participants were gradually reintroduced to normal foods, while withdrawing from the VLED products. An individual diet plan (estimated energy requirements (Ereq) at Wk9: 1690 ± 407 kcal/day) was prescribed by a trained dietician tailored to individual energy requirements (measured resting metabolic rate (RMR; Wk9 (1339 ± 252 kcal/day) × (physical activity level (PAL)) (extracted from individual physical activity monitors (BodyMedia®, SenseWear, Pittsburgh, USA), with 15–20% protein, 20–30% fat, and 50–60% carbohydrate, aimed at weight stabilisation [24]. VLED products ceased at the end of week 10.

Participants were asked not to change their PA levels during this phase of the study. To check for compliance, participants wore SenseWear armbands for 7-days at baseline and at weeks 9 and 13 (Wk9 and Wk13). Data were considered valid if the participants wore the device for ≥4 days, including at least 1 weekend day and >95% of the time [25].

#### Weight maintenance phase

A 1Y follow-up program aimed at WL maintenance was offered from Wk13. The diet plan provided at week 9 was revised at week 13 by a trained dietician having into account individual energy requirements (measured RMR (1584 ± 285 kcal/day) × PAL at Wk13) and designed for weight maintenance (Ereq at Wk13: 2088 ± 520 kcal/day). The multidisciplinary follow-up program included regular individual and group based sessions, focusing on nutritional counselling, increased PA levels and cognitive behavioural therapy. A dietitian was present in all group meetings and participants had an individual consultation with a dietitian (1 h) every other month. Participants were also asked to wear SenseWear armbands at 6 and at 12 months to record PA levels.

### Data collection

The following measurements were conducted at baseline, Wk13 and 1Y follow-up.

#### Body weight and composition

Air-displacement plethysmography (BodPod, COSMED, Italy) was used, while participants were in the fasting state.

#### Appetite measurements

Subjective appetite feelings (hunger, fullness, desire to eat (DTE) and prospective food consumption (PFC)) were measured with a 10-cm visual analogue scale (VAS) [26], and blood samples for the analysis of appetite-related hormones (active ghrelin (AG), active glucagon-like peptide 1 (GLP-1), total peptide YY (PYY), cholecystokinin (CCK), and insulin) collected in fasting and every 30 min after a standardised breakfast (600 kcal: 17% protein, 35% fat, and 48% carbohydrate) for 2.5 h. The breakfast consisted of 75 oat-bread (whole grain), 5 g butter, 40 g strawberry jam, 35 g cheese (38 E% fat) and 250 ml low fat (1.2 E% fat) milk. The satiety quotient (SQ) for each appetite sensation, as well as an average estimate, was calculated at each assessment time point [27].

Plasma samples were analysed for AG, active GLP-1, total PYY, CCK and insulin using a Human Metabolic Hormone Magnetic Bead Panel (LINCOplex Kit, Millipore) and CCK using an” in-house” RIA method [28] (intra-and inter-assay CV were for AG, active GLP-1 and PYY <10% and <20%; insulin <10% and <15% and CCK <5% and <15%, respectively. Blood was collected into EDTA-coated tubes. Around 1 ml of full blood was then transferred into a micro tube and 20 μl mixture of inhibitors (10 μl of Pefabloc (Roche Diagnostic, Germany) +10 μl DPP-IV (Merck Millipore, Germany)) added. For CCK analysis, aprotinin (DSM, Coatech AB, Kaiseraugst, Switzerland) (500 KIU/ml whole blood) was added to the EDTA tube. Samples were then centrifuged at 3200 rpm for 10 min at 18 °C and the plasma frozen at −80 °C until further analysis. Samples were analysed when all time points were available within the same participant (average 1 year). The analysis was performed by the same technician, except for CCK which was analysed at Prof. Rehfelds lab (Rigshospitalet, University of Copenhagen, Copenhagen, Denmark).

### Statistical analysis

Statistical analysis was performed with SPSS version 22 (SPSS Inc., Chicago, IL), and data presented as mean ± SEM, except for baseline anthropometric data at baseline, where mean ± SD was used. Statistical significance was set at *P* < 0.05. Data were analysed using linear mixed-effects models, with restricted maximum-likelihood estimation, including fixed effects for time and sex, and their interaction. Bonferroni correction was used for post hoc pairwise comparisons. Average values for appetite ratings and plasma concentrations of appetite-related hormones refer to the average of all time points (fasting and postprandial). Participants with data at 1Y were considered completers and kept in the analysis. All the analysis was done for completers, except for changes in body weight where an intention to treat analysis with baseline values carried forward was used. The Benjamini–Hochberg method, which controls for the false discovery rate [29] was used to adjust for the large number of outcome variables. The association between changes in subjective and objective appetite markers (both at Wk13 and 1Y), between the magnitude of WL (both at Wk13 and 1Y) and the respective changes in both subjective and objective appetite markers and between changes in appetite at Wk13 and WL maintenance at 1Y were investigated with Pearson or Spearmen correlation.

## Results

### Participants

In total 100 (55 females) participants fulfilled the study entry criteria and started the study. Of those, 95 participants completed the 8-week VLED (2 did not tolerate the VLED, 1 was excluded due to consumption of extra foods, 1 withdrew for personal reasons and 1 was lost to follow-up), 94 completed Wk 13 measurements (1 withdrew due to family illness) and 71 (41 females) completed the full 1Y (8 withdrew due to own or family related illness, 3 due to work constraints making it difficult to return for measurements, 2 were excluded due to non-compliance as they had started a new VLED, and 10 were lost to follow-up).

Baseline characteristics of the participants who started and completed the study are presented in Table 1. There were no significant differences in any baseline measurement between those who completed and those who did not complete the study. Moreover, no differences were seen between completers and non-completers regarding changes in appetite with WL (Wk13), even though completers lost more weight at Wk13 (20.0 ± 5.0 vs 16.8 ± 4.4 kg, *P* < 0.05). Females were older (*P* < 0.05), lighter (*P* < 0.001) and had a higher FM (%) than males (*P* < 0.001), but there was no difference in BMI between sexes.

**Table 1:** Baseline characteristics of the participants

	All (*N*=100)			Completers (*N*=71)			
	All (*N*=100)	Males (*n*=45)	Females (*n*=55)	All (*N*=71)	Males (*n*=30)	Females (*n*=41)	*P*-value
Age (year)	42.5 ± 9.7	39.7 ± 9.1	44.8 ± 9.7**	43.4 ± 9.4	40.3 ± 9.2	45.6 ± 9.0*	0.140
Weight (kg)	110.3 ± 18.4	120.1 ± 19.6***	102.3 ± 12.8	109.1 ± 18.7	120.4 ± 19.7***	100.9 ± 12.9	0.313
BMI (kg/m)	36.7 ± 4.2	36.6 ± 4.9	36.7 ± 3.5	36.4 ± 4.0	36.6 ± 4.7	36.2 ± 3.5	0.274
FM %	44.1 ± 6.4	39.3 ± 5.7	47.9 ± 3.9***	44.0 ± 6.4	39.2 ± 6.2	47.3 ± 4.0***	0.849

Values are mean ± SD

*BMI* Body-mass index (calculated as the weight in kg divided by the square of the height in metres). *FM* Fat mass

*P*-values are for comparisons between all participants and completers. Symbols denote significant differences between sexes in each group

*P < 0.05, **P < 0.01 and ***P < 0.001

No changes in total PA duration or time spent in light, moderate or vigorous activities were measured during the WL phase. Time spent in vigorous activities was significantly increased at 6 months in all participants and females (*P* < 0.01 and *P* < 0.05, respectively), but returned to baseline levels at 1Y for all groups. Steps/day were significantly increased at 6 months in all participants (*P* < 0.01) and at 1Y in males only (*P* < 0.05). See Supplementary Table 1.

### Body weight and composition

**Fig. 1:**
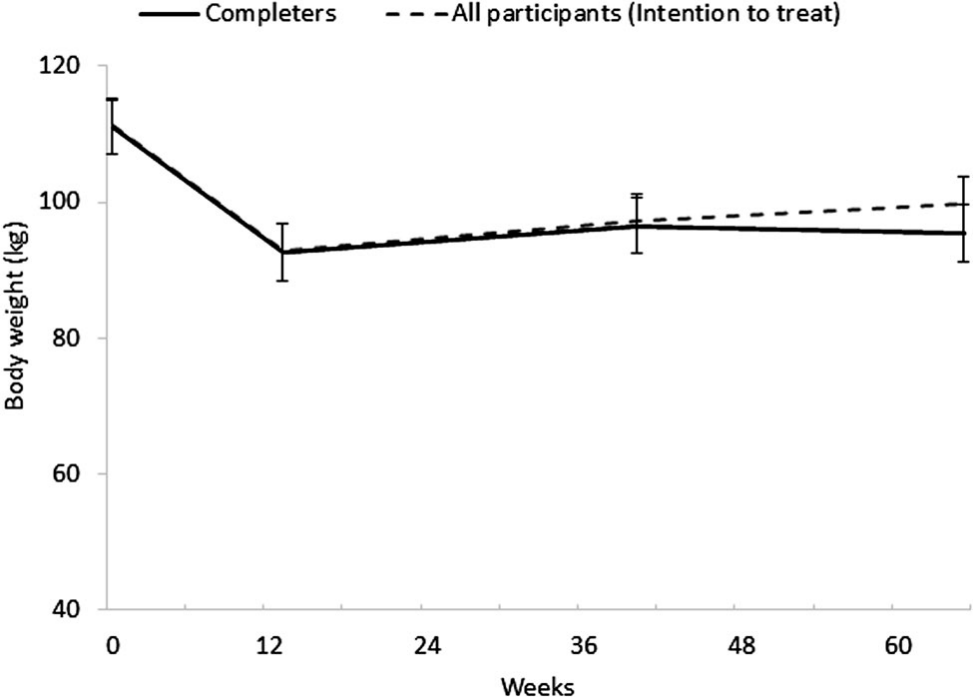
Body weight in all participants (intention to treat analysis) and completers over time. Results presented as mean ± SEM

Changes in body weight are reported in Fig. 1. Mean WL at Wk13 was 16% (−18 ± 1 kg), and this was maintained at 1Y follow-up in completers: 15% WL (−16 ± 1 kg, *P* < 0.001). The intention to treat analysis revealed an increase in body weight from Wk13 to 1Y in all participants (6 ± 1 kg, *P* < 0.001), but body weight at 1Y was still significant lower than baseline (-11 ± 1 kg, *P* < 0.001).

FM (%) was significantly decreased at Wk13 in all participants, males and females (−9 ± 1%, −11 ± 1% and −7 ± 1%, respectively, *P* < 0.001 for all groups) and remained lower than baseline at 1Y (−9 ± 1%, −9 ± 1% and −8 ± 1%, respectively, *P* < 0.001 for all groups). The changes in absolute FM over time were not statistical significant different between sexes.

### Appetite feelings

**Fig. 2:**
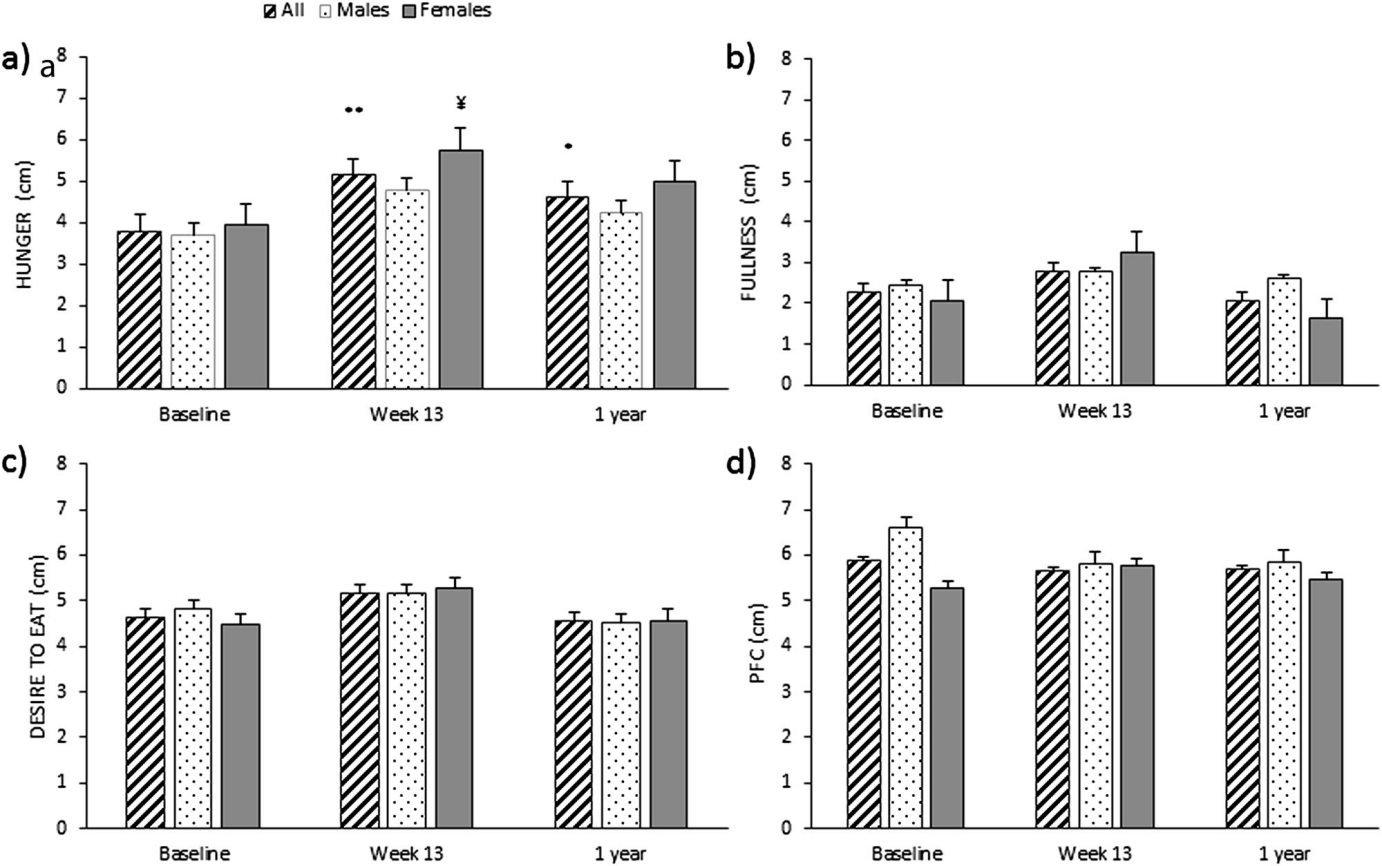
Subjective feelings of hunger (**a**), fullness (**b**), desire to eat (**c**), and prospective food consumption (PFC) (**d**) in fasting, over time, in all participants, males and females. Results presented as estimated marginal means ± SEM. Symbols denote significant differences from baseline in all participants: ^**^P < 0.01 and ^*^P < 0.05, and females: ^¥^*P* < 0.05

Hunger ratings in fasting were increased at Wk13 in all participants (38%) and females (*P* < 0.01 and *P* < 0.05, respectively), and this was sustained at 1Y follow-up in all participants (22%) (*P* < 0.05). No significant change overtime were found for rating of fullness, DTE or PFC in fasting (see Fig. 2). Females had overall significant lower ratings of PFC in fasting than males (5.4 ± 0.2 vs.6.1 ± 0.2 cm, *P* < 0.05).

**Fig. 3:**
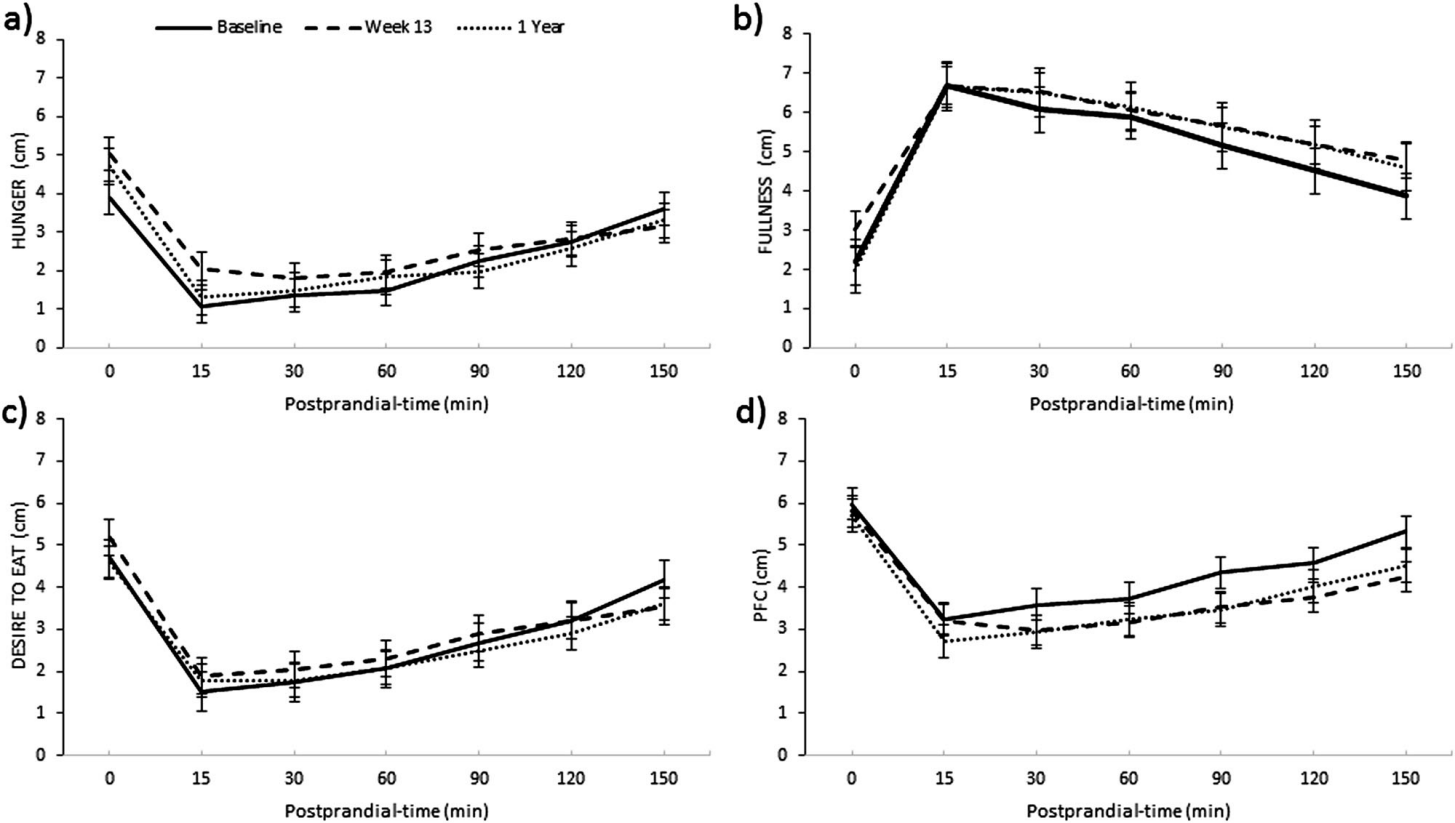
Mean fasting and postprandial ratings of hunger (**a**), fullness (**b**), desire to eat (**c**), and prospective food consumption (PFC) (**d**) in all participants over time. Results presented as estimated marginal means ± SEM

Mean ratings of hunger were significantly increased in all participants and males at Wk13 (*P* < 0.01 and *P* < 0.05, respectively) compared to baseline, but this was not sustained at 1Y follow-up. Mean fullness ratings were significantly increased in all participants (10%) and males at Wk13 (*P* < 0.01 and *P* < 0.05, respectively), and this was sustained at 1Y follow-up in all participants (6%) (*P* < 0.01), but not in males. Females, had a significant increase in mean rating of fullness at 1Y follow-up only (*P* < 0.05). Mean ratings of PFC were significantly reduced in all participants and males at Wk13 (*P* < 0.001 and *P* < 0.001, respectively) and this was sustained at 1Y follow-up (*P* < 0.001, for both) (see Fig. 3). Females experienced a significant reduction in ratings of PFC at 1Y only (*P* < 0.05), and had significant lower overall rating of DTE (2.3 ± 0.2 vs. 3.3 ± 0.3 cm, *P* < 0.05) and PFC (3.4 ± 0.3 vs. 4.6 ± 0.3 cm, *P* < 0.01), compared to males. See Supplementary Table 2A.

A significant increase in SQ hunger was seen at Wk13 and this was sustained at 1Y in all participants (*P* < 0.01 and *P* < 0.05, respectively) and females (*P* < 0.05, for both). See Supplementary Table 3.

### Appetite-related hormones

**Fig. 4:**
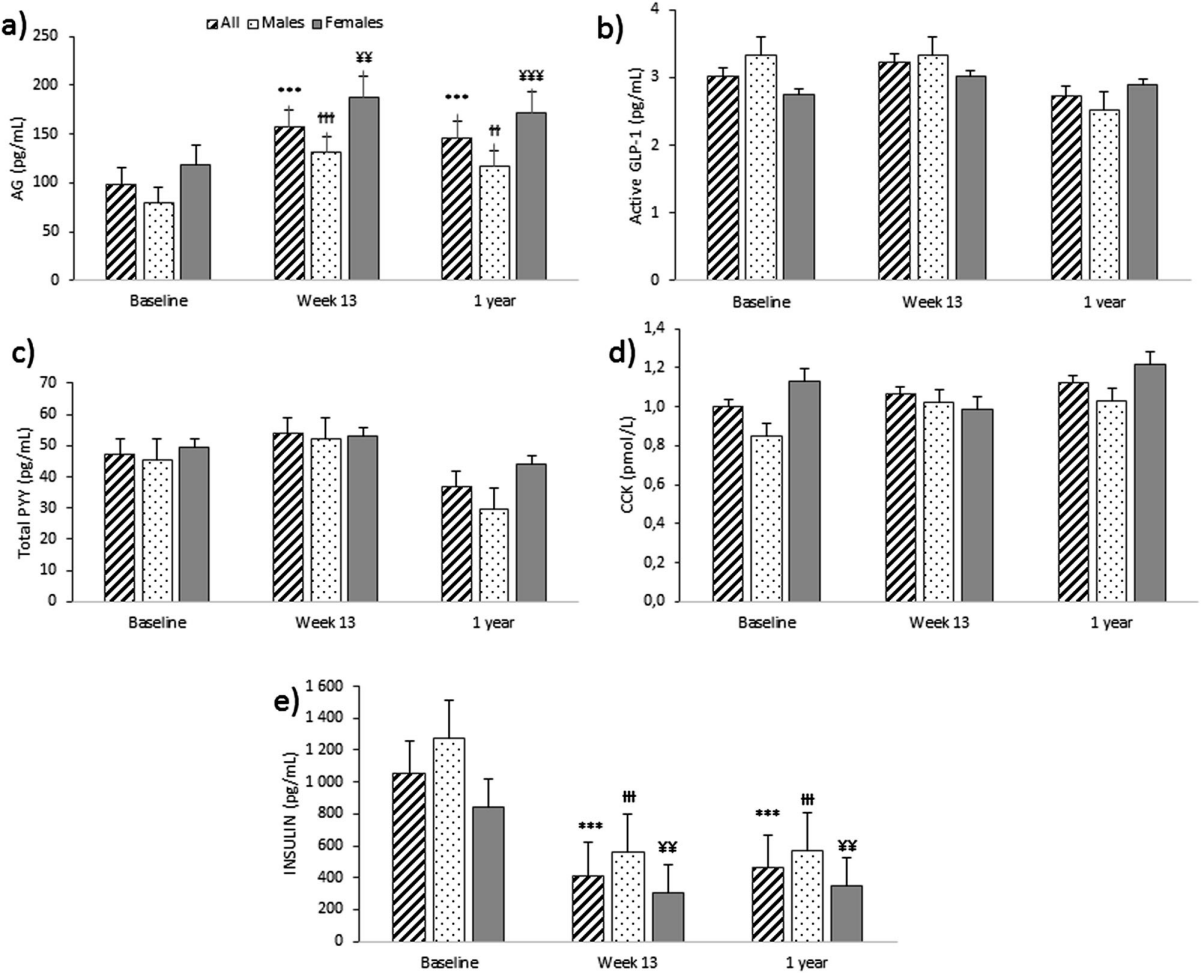
Basal plasma concentrations of appetite-related hormones: active ghrelin (AG) (**a**), active glukagon like peptide-1 (GLP-1) (**b**), total peptide YY (total PYY) (**c**), cholecystokinin  (CCK) (**d**) and insulin (**e**), over time in all participants, males and females. Results presented as estimated marginal means ± SEM. Symbols denote significant differences from baseline in all participants: ^***^P < 0.001, males: ^ƗƗƗ^*P* < 0.001 and ^ƗƗ^*P* < 0.01 and females: ^¥¥¥^*P* < 0.001 and ^¥¥^*P* < 0.05

Basal concentration of AG was significantly increased in all participants, males and females at Wk13 (*P* < 0.001, *P* < 0.001, and *P* < 0.01, respectively) and this was sustained at 1Y follow-up (*P* < 0.001, *P* < 0.01, and *P* < 0.001, respectively). Basal insulin concentration was significantly reduced in all participants, males and females at Wk13 (*P* < 0.01, *P* < 0.001, and *P* < 0.01, respectively) and this was sustained at 1Y (*P* < 0.001, for all groups). Females had significant overall lower basal concentration of insulin (485 ± 66 vs. 803 ± 69 pg/mL, *P* < 0.01) than males (see Fig. 4).

**Fig. 5:**
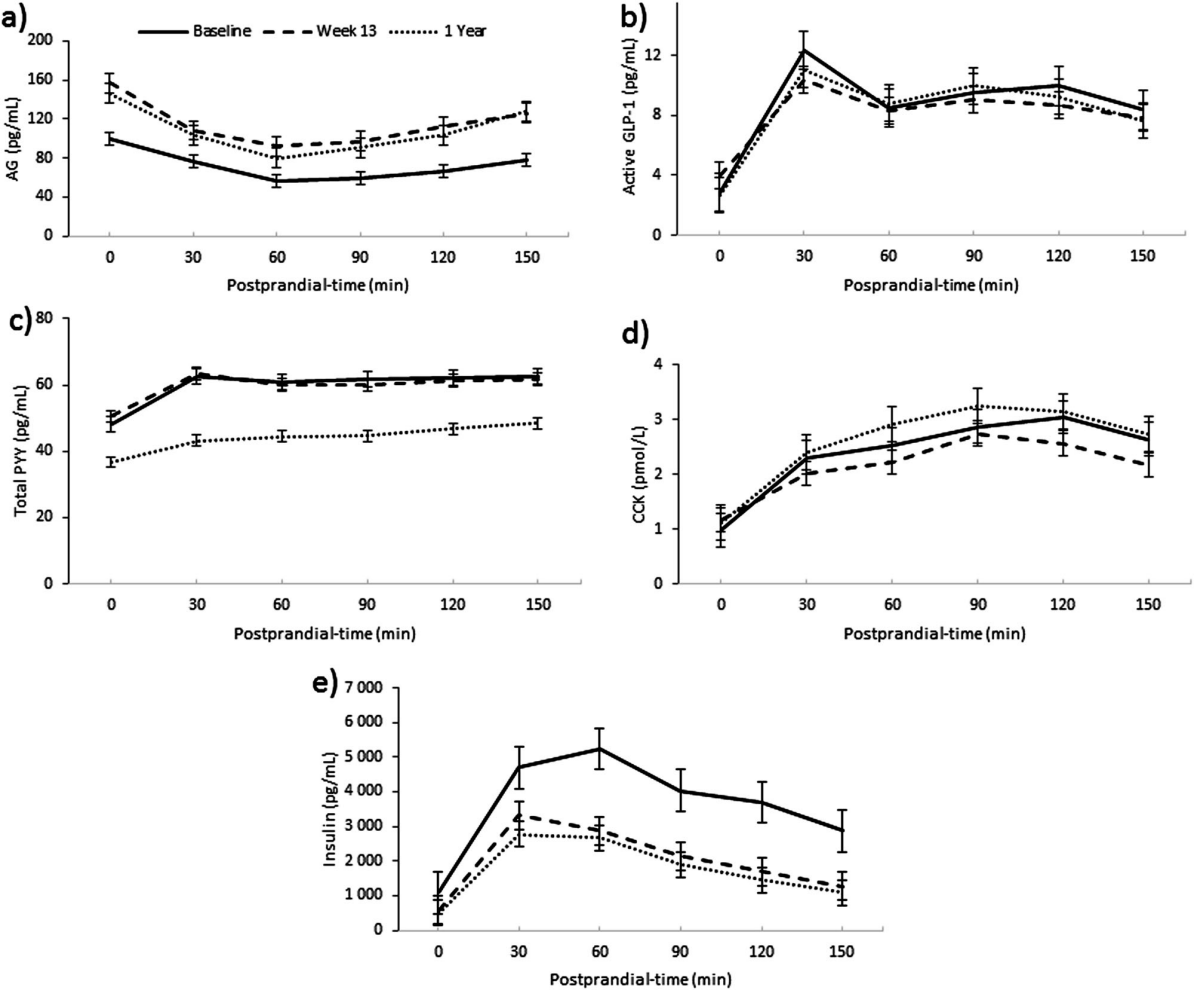
Mean basal and postprandial plasma concentrations of appetite-related hormones; **a)** active ghrelin (AG) (**a**), **b)** active glucagon-like peptide-1 (active  GLP-1) (**b**), **c)** total peptide YY (total  PYY) (**c**), **d)** cholecystokinin(CCK) (**d)**, and **e** insulin (**e**), for all participants over time. Results presented as estimated marginal means ± SEM

Mean AG plasma concentration was significantly increased at Wk13 in all participants, males and females (*P* < 0.001, for all groups) and this was sustained at 1Y follow-up (*P* < 0.001, for all groups). Mean total PYY concentration did not change significantly by Wk13, but was significantly lower than baseline at 1Y follow-up in all participants, males and females (*P* < 0.001, *P* < 0.001, and *P* < 0.01, respectively). There was a significant reduction in mean CCK concentrations in all participants at Wk13 (*P* < 0.01), but an increase at 1Y follow-up, compared with baseline, in all participants and males (*P* < 0.01, for both). There was a significant reduction in mean insulin concentration in all participants, males and females at Wk13 (*P* < 0.001, for all groups), which was sustained at 1Y (*P* < 0.001, for all groups) (see Fig. 5). See Supplementary Table 2B.

### Correlation analysis

No significant correlation was found between changes in subjective appetite feelings and changes in the plasma concentrations of appetite-related hormones at any time point (either at W13 or 1Y). The larger the WL at 1Y, the larger was the increase seen in basal and postprandial concentration of AG (*r* = −0.455 and *r* = −0.566, respectively, *P* < 0.001, for both). The opposite was seen for insulin, with a larger reduction in basal and postprandial insulin plasma concentrations as the magnitude of WL increased at 1Y (*r* = 0.282 and *r* = 0.277, respectively, *P* < 0.05 for both).

No significant association was found between the changes in appetite (namely increased hunger and AG plasma concentrations) seen with WL (Wk13) and WL maintenance/relapse at 1Y.

## Discussion

In this longitudinal study, a 16% WL at Wk13 was sufficient to induce significant health benefits [30, 31], and was associated with an increase in hunger, while postprandial feelings of fullness and SQ hunger were increased and PFC reduced. There was also a significant increase in basal and postprandial AG concentrations, a reduction in postprandial CCK and in basal and postprandial insulin. At 1Y, and with sustained WL (15%), fasting hunger, SQ hunger and postprandial fullness ratings were still increased and postprandial PFC reduced. Basal and postprandial AG remained increased and insulin reduced, while postprandial CCK was increased and PYY decreased at 1Y follow-up, compared to baseline.

Few studies have been performed on the long-term sustainability of changes in appetite with WL and, to date, the results have been contradictory [15, 20, 21]. Sumithran et al. (2011) [15], in a similar longitudinal study, reported a sustained increase in postprandial hunger and DTE ratings, and AG plasma concentrations and a reduction in the postprandial concentration of total PYY and CCK at 1Y follow-up [15]. However, the sample was composed of predominately post-menopausal women (68%) and participants experienced an average 50% weight regain, after the initial 14% WL. Iepsen et al. [21], using a similar study design, reported an increase in the plasma concentration of total ghrelin (both in the fasting and postprandial states), but surprisingly, an increase in total GLP-1 and PYY_3–36_ postprandial concentrations with a sustained 13% WL at 1Y follow-up. Unfortunately, they did not measure changes in subjective feelings of appetite. Adam et al. [20], on the other hand, reported an increase in the postprandial concentrations of active GLP-1 after an 8% WL, which was not sustained after a 12-week WL maintenance period. The present study is the largest evaluating the impact of sustained WL maintenance, employing both subjective and objective appetite markers, and offers a perspective of sex being a mediator of outcome.

Even though ours and other studies tend to consistently show a sustained increase in subjective feelings of hunger, when using VAS, combined with an increase in ghrelin (either active or total) with long-term WL [15, 21], changes in postprandial fullness ratings and plasma concentrations of satiety peptides remain controversial [15, 21]. Differences in the magnitude of WL and its sustainability, hormonal fractions measured and methods of analysis of gut peptides [32–34] are likely to contribute to this inconsistent picture alongside most studies having mixed gender. Moreover, SQ hunger increased with WL, indicating a larger reduction in hunger after the same test meal, which is likely to reflect more accurate appetite sensation responses [35].

The increase in postprandial fullness with WL and WL maintenance is a novel. Sumithran et al. [15] reported no change in fullness either with a 14% WL at Wk 10 or with a 7% WL maintenance at 1Y follow-up in 34 overweight and obese individuals. Adam et al. [20] reported a similar outcome after an 8% WL, both acutely and after 12 weeks of WL maintenance in 32 overweight and obese individuals. Inconsistencies may be due to differences in the magnitude of WL, baseline participant characteristics and sample size. In our study, an initial 16% WL was achieved, and this was sustained at 1Y (15% WL) in 71 obese individuals, which is a much larger WL and sample size compared with the other available studies [15, 20]. In a study by Delgado-Aros et al. [36], a higher BMI was associated with decreased postprandial fullness, which may explain why a so large WL, and concomitant BMI reduction, in our study led to increased fullness, when compared with baseline ratings. Even though studies on the impact of BMI on gastric capacity have not been consistent [37], a reduction in fasting gastric capacity has been reported after ≥5% WL in individuals with obesity [38]. It remains speculative if changes in gastric capacity with WL contributed to increased postprandial fullness in the present study. Increased fullness after acute and sustained WL is unlikely to be explained by changes in CCK secretion, given that a reduction in CCK secretion was measured at Wk13, while an increase was seen with sustained WL at 1Y follow-up. Another explanation for the increased fullness with WL may be an increased postprandial secretion GLP-1 and PYY. Even though we saw no change in active GLP-1, and a reduction in total PYY plasma concentrations, others have shown that a large WL (14–15%) leads to an increase in the secretion of both total GLP-1 [21, 39] and PYY_3–36_ [21]. Finally, it is possible that increased fullness reflects the larger relative energy load of the test meal after WL.

To date, there is a dearth of information on the impact of WL on CCK plasma concentrations. The available evidence suggests that acute and rapid WL results in a reduction in postprandial concentrations of CCK [15, 40], which is consistent with our results, and probably reflects a lower stimulation due to less food (and fat) intake. It remains speculative why in the present study an increase in postprandial concentrations of CCK was seen with sustained WL at 1Y follow-up, while in Sumithran’s study [15], with a similar design (8 weeks of VLED followed by refeeding and 1 year follow-up) and methodology (same RIA protocol for CCK analysis), a reduction was reported. However, due to continued dietetic support we achieved a 15% sustained WL at 1Y follow-up, while Sumithran had a 50% weight regain, with only a 7% WL at 1Y follow-up. This could have had an impact on CCK concentrations, and the increased postprandial CCK secretion may reflect a long-term adaptation to substantial WL, but that requires further substantiation.

The lack of association between subjective appetite feelings and the plasma concentration of appetite-related hormones seen in this study is not new [41, 42] and probably reflects the complexity of the appetite control system and the fact that changes in appetite feelings are unlikely to be attributable to alterations in a single hormone.

It has long been suggested that the increased hunger and reduced satiety seen after WL are part of a compensatory response that tries to bring body weight back to its set point [8, 43–45]. However, the findings from the present study: increased fasting hunger and basal AG plasma concentrations, as well as increased postprandial fullness, AG and CCK in response to large sustained WL may suggest otherwise. It is well known that obese individuals have lower plasma concentration of ghrelin in fasting [46] and a blunted postprandial secretion of total GLP-1 [39, 47], active GLP-1 [48], total PYY [48, 49], and CCK [50] and lower ghrelin postprandial suppression [46, 48]. Therefore, our overall findings, with the exception of PYY, could reflect a normalisation of appetite markers towards those seen in healthy-weight individuals. This is supported by Verdich et al. [39], who showed that postprandial total GLP-1 concentration increased after a 19 kg WL toward levels seen in a control normal-weight group. WL also leads to a reduction in TEE proportional to the new reduced body weight, even though some individuals may experience a larger than predicted reduction—a mechanism known as adaptive thermogenesis (AT) [10]. With, the exception of AT, which seems to occur in only some individuals, the changes in appetite and energy expenditure seen with WL could, therefore, be seen as a normalisation towards a lower body weight and not a compensatory mechanism that drives relapse. This new hypothesis is supported by the fact that neither us, nor Sumithran et al. [15] have reported any association between the changes in appetite seen with WL and long-term relapse at 1 year follow-up.

This study revealed several sex differences in the changes in appetite seen overtime with WL and WL maintenance. Hunger ratings in fasting were increased at Wk13 in females only, while mean postprandial ratings of hunger and fullness were significantly increased, and PFC reduced, in males only at Wk13, and postprandial fullness was increased in females only at 1Y follow-up. Moreover, mean postprandial CCK plasma concentrations did not change at any time point in females, while in males there was an increase at 1Y follow-up, compared to baseline. The fact that postprandial fullness was increased at 1Y follow-up in females only may reflect the fact that in males there was a tendency towards weight regain from Wk13 to 1Y, while females continued to lose weight over time. More studies, with larger sample sizes and equal sex distribution, are needed to fully ascertain the potential modulating effect of sex on the changes in appetite seen with WL and WL maintenance and the explanatory mechanisms behind it.

This study has several strengths. First, it is the largest longitudinal study to examine changes in appetite with sustained WL. Second, it included both objective and subjective markers of appetite. Third, the participants were able to maintain their body weight at 1Y follow-up (compared with Wk13), probably due to on-going and tailored advice provided by dieticians. Finally, both males and females were included in the study in similar numbers. There are also some limitations. The multiplex assay used for the measurements of appetite hormones (except for CCK) is likely to result in less accurate and precise measurements compared with optimised assays for each individual hormone. The fact that the same type of test meal was given to all participants, regardless of their Ereq, constitutes a limitation. Females consumed a larger relative energy load compared with males and the same test meal represented a larger energy load with progressive WL. However, if we had adjusted the test meal accordion to Ereq (smaller meals in females compared with females and after WL) the appetite response would be blunted, because the nutrient stimuli would also be reduced, independently of the effect of sex or WL on appetite. This study was not powered to examine sex differences per se, so we are unable to draw firm conclusions about sex differences in responses to WL.

Our findings have some important practical implications. Patients with obesity who have lost and maintained significant amounts of weight via dieting, and benefited in terms of metabolic and overall health markers [30, 31], should expect a sustained increase in hunger feelings in the fasting state and to be prepared for these feelings to occur. This increased drive to eat in fasting may impact on food selection, eating rate and total energy intake, despite increased postprandial fullness, and thus lead to positive energy imbalance and increase the risk of weight regain. Health professionals working with this patient group, should be aware of the sustained increase in the drive to eat in the fasting state and help individuals develop management strategies to reduce the risk of overeating. However, the changes in appetite seen with WL (increased hunger and AG) were not associated with long-term relapse, which likely reflects the complexity of body weight regulation [51].

## Conclusions

With a 15% sustained WL at 1Y follow-up, the drive to eat in the fasted state is increased, which may, despite increased postprandial fullness, drive overeating. Some sex differences were revealed, but larger studies are needed to support these findings. Future studies should evaluate if changes in appetite markers with WL are part of a compensatory response or a simple normalisation towards healthy-weight values, its relationship with actual food intake and its real impact on long-term WL maintenance.

## Electronic supplementary material

Supplementery Table1

Supplementery Table 2 A

Supplementery Table 2 B

Suplementery Table 3

Supplementery Figure I

Supplementery Figure I ledgend
