# Supplementary material for: Investigation of the long-term sustainability of changes in appetite after weight loss
Source: Int J Obes (Lond). 2018 Jun 21;42(8):1489–99. doi: 10.1038/s41366-018-0119-9 (PMC6113192; doi:10.1038/s41366-018-0119-9)
Supplement: Supplementary file 1 — Supplementery Table1 [file 41366_2018_119_MOESM1_ESM.pdf]

Supplementary table I. Physical activity levels over time in all participants, males and females

	Baseline	Week 13	6 months	1 year
**Average MET (kcal/kg/hour)**				
All	1.20 ± 0.02	1.33 ± 0.030***	1.34 ± 0.030***	1.29 ± 0.02**
Males	1.28 ± 0.04	1.42 ± 0.04ǂǂ	1.42 ± 0.04ǂǂ	1.37 ± 0.04
Females	1.13 ± 0.03	1.23 ± 0.05	1.26 ± 0.05¥	1.21 ± 0.03¥¥
**SedTime (min/day)**				
All	1134.98 ± 15.43	1111.33 ± 18.68	1105.94 ± 18.14	1119.15 ± 15.25
Males	1113.23 ± 23.66	1078.46 ± 24.29	1096.95 ± 24.60	1101.46 ± 23.46
Females	1156.05 ± 20.01	1163.07 ± 31.53	1109.06 ± 27.67	1137.60 ± 19.74
**LightAct (min/day)**				
All	216.07 ± 12.29	241.84 ± 15.14	232.29 ± 14.67	230.90 ± 12.13
Males	212.60 ± 18.79	250.81 ± 19.34	22965 ± 19.61	214.17 ± 18.61
Females	219.96 ± 15.86	210.36 ± 25.80	230.80 ± 22.50	243.34 ± 15.62
**ModAct (min/day)**				
All	69.90 ± 7.42	74.73 ± 9.44	96.06 ± 9.10	82.82 ± 7.30
Males	92.18 ± 11.44	95.32 ± 11.83	126.94 ± 12.03	112.23 ± 11.30
Females	46.49 ± 9.62	61.74 ± 16.55	62.37 ± 14.27	54.54 ± 9.45
**VigAct (min/day)**				
All	0.81 ± 1.16	4.43 ± 1.52	6.71 ± 1.46**	3.48 ± 1.14
Males	1.18 ± 1.80	6.44 ± 1.86	6.22 ± 1.90	4.97 ± 1.77
Females	0.31 ± 1.51	0.84 ± 2.69	7.95 ± 2.30¥	2.20 ± 1.48
**TotalPA (min/day)**				
All	288.17 ± 13.50	315.35 ± 16.28	326.87 ± 15.82	292.61 ± 13.35
Males	305.13 ± 20.70	349.85 ± 21.25	350.11 ± 21.51	322.21 ± 20.53
Females	268.76 ± 17.51	266.80 ± 27.47	303.51 ± 24.14	264.10 ± 17.29
**StepsDay**				
All	6650.35 ± 389.73	7111.89 ± 468.70	8600.74 ± 455.75**	7883.77 ± 385.49
Males	6198.86 ± 598.77	6915.94 ± 614.45	8553.78 ± 621.87	7820.95 ± 593.96ǂ
Females	7026.84 ± 506.58	7276.78 ± 791.01	8528.31 ± 695.72	7984.04 ± 500.14

Results presented as estimated marginal means±SEM. MET; Metabolic Equivalent of Task. Act; activity. Mod; moderate. Vig; vigorous/very vigorous. PA; physical activity. Min; minutes. Symbols denote significant differences from baseline (for all participants ***P<0.001 and **P<0.01, males ǂǂP<0.01 and ǂP<0.05, and females ¥¥P<0.01 and ¥P<0.05).
