# Supplementary material for: Investigation of the long-term sustainability of changes in appetite after weight loss
Source: Int J Obes (Lond). 2018 Jun 21;42(8):1489–99. doi: 10.1038/s41366-018-0119-9 (PMC6113192; doi:10.1038/s41366-018-0119-9)
Supplement: Supplementary file 2 — Supplementery Table 2 A [file 41366_2018_119_MOESM2_ESM.docx]

|  |  |  |  |  |  | |  |  |  |  | |  | |  | | |  |  |  |  |
| --- | --- | --- | --- | --- | --- | --- | --- | --- | --- | --- | --- | --- | --- | --- | --- | --- | --- | --- | --- | --- |
| Supplementary table 2 A. Subjective feelings of appetite feelings variables at baseline and change over time in all participants. | | | | | | | | | | | | | | | | | | | | |
|  | Baseline | | | | | | | | Δ B to W13 | | | | P-value  (B to W13) | Δ B to 1 Y | | | P-value  (B to 1 Y) |  |  |  |
| *Fasting* |  | | | | |  | |  |  |  |  | |  |  |  |  |  |  |  |  |
| Hunger | 3.7 | | | | | ± | | 0**.2** | 1.4 | ± | 0.4 | | <0.01 | 0.8 | ± | 0.3 | <0.05 |  |  |  |
| Fullness | 2.3 | | | | | ± | | 0.2 | 0.8 | ± | 0.3 | | 0.081 | -0.2 | ± | 0.3 | 0.179 |  |  |  |
| DTE | 4.6 | | | | | ± | | 0.3 | 0.6 | ± | 0.4 | | 0.442 | -0.1 | ± | 0.3 | 0.240 |  |  |  |
| PFC | 5.9 | | | | | ± | | 0.3 | -0.3 | ± | 0.4 | | 0.252 | -0.2 | ± | 0.4 | 0.141 |  |  |  |
| *2.5-hour AUC* |  | | | | |  | |  |  |  |  | |  |  |  |  |  |  |  |  |
| Hunger | 347.5 | | | | | ± | | 28.3 | 51.0 | ± | 35.2 | | 0.452 | 6.5 | ± | 27.2 | 0.202 |  |  |  |
| Fullness | 872.5 | | | | | ± | | 31.0 | 74.2 | ± | 38.6 | | 0.171 | 61.2 | ± | 29.8 | 0.126 |  |  |  |
| DTE | 424.5 | | | | | ± | | 32.3 | 22.4 | ± | 37.6 | | 0.240 | -15.5 | ± | 28.4 | 0.244 |  |  |  |
| PFC | 683.4 | | | | | ± | | 39.0 | -125.8 | ± | 54.7 | | 0.07 | -99.5 | ± | 39.4 | <0.05 |  |  |  |
|  |  | | | | |  | |  |  |  |  | |  |  |  |  |  |  |  |  |
| *Average* |  | | | | |  | |  |  |  |  | |  |  |  |  |  |  |  |  |
| Hunger | 2.4 | | | | | ± | | 0.2 | 0.4 | ± | 0.2 | | <0.05 | 0.1 | ± | 0.1 | 0.990 |  |  |  |
| Fullness | 4.9 | | | | | ± | | 0.2 | 0.5 | ± | 0.2 | | <0.01 | 0.3 | ± | 0.1 | <0.01 |  |  |  |
| DTE | 2.9 | | | | | ± | | 0.2 | 0.1 | ± | 0.1 | | 0.951 | -0.1 | ± | 0.1 | 0.562 |  |  |  |
| PFC | 4.4 | | | | | ± | | 0.2 | -0.6 | ± | 0.2 | | <0.001 | -0.6 | ± | 0.1 | <0.001 |  |  |  |

Results presented as estimated marginal means±SEM. DTE: desire to eat. PFC: Prospective food consumption.

AUC: total area under the curve. Symbols denote significant differences from baseline ***P<0.001, **P<0.01 and *P<0.05.
