# Supplementary material for: Investigation of the long-term sustainability of changes in appetite after weight loss
Source: Int J Obes (Lond). 2018 Jun 21;42(8):1489–99. doi: 10.1038/s41366-018-0119-9 (PMC6113192; doi:10.1038/s41366-018-0119-9)
Supplement: Supplementary file 3 — Supplementery Table 2 B [file 41366_2018_119_MOESM3_ESM.docx]

| Supplementary table 2B. Appetite related hormones at baseline and changes over time in all participants. | | | | | | | | | | | | | | | | | | | | | |  |  |  |  |  |  |  |  |  |
| --- | --- | --- | --- | --- | --- | --- | --- | --- | --- | --- | --- | --- | --- | --- | --- | --- | --- | --- | --- | --- | --- | --- | --- | --- | --- | --- | --- | --- | --- | --- |
|  | | Baseline | | | | Δ B to W13 | | | | | | P-value  (B to W13) | | | Δ B to 1Y | | | | | P-value (B to 1Y) | |  |  |  |  |  |  |  |  |  |
| *Fasting* |  | | | | | | | |  |  | | |  | | | | |  | | | | |  |  |  |  |  |  |  |  |
| Ghrelin | 98.1 | | ± | 11.4 | 59.0 | | ± | 10.5 | | | <0.001 | | | 47.1 | | ± | 8.2 | | <0.001 | |  |  |  |  |  |  |  |  |  |  |
| GLP-1 | 3.0 | | ± | 0.5 | 0.2 | | ± | 0.6 | | | 0.240 | | | -0.3 | | ± | 0.5 | | 0.164 | |  |  |  |  |  |  |  |  |  |  |
| PYY | 47.1 | | ± | 6.5 | 6.8 | | ± | 6.9 | | | 0.978 | | | -10.1 | | ± | 5.4 | | 0.194 | |  |  |  |  |  |  |  |  |  |  |
| CCK | 1.0 | | ± | 0.1 | 0.1 | | ± | 0.2 | | | 0.313 | | | 0.1 | | ± | 0.1 | | 0.106 | |  |  |  |  |  |  |  |  |  |  |
| Insulin | 1053.5 | | ± | 57.2 | -640.0 | | ± | 77.4 | | | <0.001 | | | -588.1 | | ± | 60.3 | | <0.001 | |  |  |  |  |  |  |  |  |  |  |
|  |  | |  |  |  | |  |  | | |  | | |  | |  |  | |  | |  |  |  |  |  |  |  |  |  |  |
| *2.5-hour AUC* |  | |  |  |  | |  |  | | |  | | |  | |  |  | |  | |  |  |  |  |  |  |  |  |  |  |
| Ghrelin | 10390 | | ± | 1188 | 5889 | | ± | 962 | | | <0.001 | | | 4935 | | ± | 752 | | <0.001 | |  |  |  |  |  |  |  |  |  |  |
| GLP-1 | 1375 | | ± | 92 | -133 | | ± | 127 | | | 0.891 | | | -61 | | ± | 99 | | 0.193 | |  |  |  |  |  |  |  |  |  |  |
| PYY | 8993 | | ± | 962 | 15 | | ± | 941 | | | 0.327 | | | -2274 | | ± | 732 | | <0.01 | |  |  |  |  |  |  |  |  |  |  |
| CCK | 375 | | ± | 17 | -41 | | ± | 19 | | | 0.111 | | | 31 | | ± | 15 | | 0.132 | |  |  |  |  |  |  |  |  |  |  |
| Insulin | 586992 | | ± | 29253 | -281129 | | ± | 37189 | | | <0.001 | | | -299002 | | ± | 28885 | | <0.001 | |  |  |  |  |  |  |  |  |  |  |
|  |  | |  |  |  | |  |  | | |  | | |  | |  |  | |  | |  |  |  |  |  |  |  |  |  |  |
| *Average* |  | |  |  |  | |  |  | | |  | | |  | |  |  | |  | |  |  |  |  |  |  |  |  |  |  |
| Ghrelin | 72.6 | | ± | 8.0 | 42.7 | | ± | 3.6 | | | <0.001 | | | 35.9 | | ± | 2.5 | | <0.001 | |  |  |  |  |  |  |  |  |  |  |
| GLP-1 | 8.6 | | ± | 0.5 | -0.6 | | ± | 0.5 | | | 0.802 | | | -0.4 | | ± | 0.4 | | 0.900 | |  |  |  |  |  |  |  |  |  |  |
| PYY | 59.7 | | ± | 5,9 | 0.2 | | ± | 2,7 | | | 0.315 | | | -15.7 | | ± | 2.0 | | <0.001 | |  |  |  |  |  |  |  |  |  |  |
| CCK | 2.4 | | ± | 0.1 | -0.3 | | ± | 0.1 | | | <0.01 | | | 0.2 | | ± | 0.1 | | <0.01 | |  |  |  |  |  |  |  |  |  |  |
| Insulin | 3599.2 | | ± | 164.0 | -1620.3 | | ± | 146.6 | | | <0.001 | | | -1872.3 | | ± | 100.4 | | <0.001 | |  |  |  |  |  |  |  |  |  |  |

Results presented as estimated marginal means±SEM. B: baseline. W13: week 13. 1Y: 1 year. AG: active ghrelin.

GLP-1: glucagon-like peptide-1. CCK: cholecystokinin. PYY: total peptide YY. AUC: total area under

the curve. Symbols denote significant differences from baseline ***P<0.001, **P<0.01 and *P<0.05.
