# Supplementary material for: Investigation of the long-term sustainability of changes in appetite after weight loss
Source: Int J Obes (Lond). 2018 Jun 21;42(8):1489–99. doi: 10.1038/s41366-018-0119-9 (PMC6113192; doi:10.1038/s41366-018-0119-9)
Supplement: Supplementary file 4 — Suplementery Table 3 [file 41366_2018_119_MOESM4_ESM.docx]

| Supplementary Table 3. Satiety quotient (SQ, mm/kcal) overtime with progressive WL in participants, males and females. | | | | | | | | | | |
| --- | --- | --- | --- | --- | --- | --- | --- | --- | --- | --- |
|  |  | All groups | | | Males | | | Females | | |
| SQ Hunger | B | 2,4 | ± | 0,3 | 1,7 | ± | 0,5 | 3,2 | ± | 0,5* |
|  | W13 | 4,2 | ± | 0,5## | 2,8 | ± | 0,6 | 6,0 | ± | 0,9**# |
|  | 1Y | 3,6 | ± | 0,3# | 2,4 | ± | 0,5 | 4,7 | ± | 0,5**# |
| SQ Fullness | B | 4,9 | ± | 0,4 | 3,8 | ± | 0,6 | 6,1 | ± | 0,5** |
|  | W13 | 4,6 | ± | 0,5 | 3,8 | ± | 0,7 | 4,7 | ± | 1,0 |
|  | 1Y | 5,2 | ± | 0,4 | 3,9 | ± | 0,6 | 6,5 | ± | 0,5** |
| SQDTE | B | 2,9 | ± | 0,3 | 2,5 | ± | 0,5 | 3,4 | ± | 0,4 |
|  | W13 | 3,8 | ± | 0,4 | 2,9 | ± | 0,5 | 5,1 | ± | 0,8* |
|  | 1Y | 2,9 | ± | 0,3 | 2,3 | ± | 0,5 | 3,6 | ± | 0,4* |
| SQ PFC | B | 2,7 | ± | 0,5 | 2,9 | ± | 0,7 | 2,8 | ± | 0,6 |
|  | W13 | 3,5 | ± | 0,7 | 2,6 | ± | 0,8 | 5,2 | ± | 1,2 |
|  | 1Y | 3,1 | ± | 0,5 | 2,5 | ± | 0,7 | 3,6 | ± | 0,6 |
| Average SQ |  |  |  |  |  |  |  |  |  |  |
| SQB |  | 3,2 | ± | 0,3 | 2,7 | ± | 0,4 | 3,9 | ± | 0,4* |
| SQW13 |  | 4,0 | ± | 0,4 | 3,0 | ± | 0,4 | 5,3 | ± | 0,7** |
| SQ1Y |  | 3,7 | ± | 0,3 | 2,8 | ± | 0,4 | 4,6 | ± | 0,4** |
| Results expressed as estimated marginal means±SEM. PFC: prospective food consumption. DTE: desire to eat. Significant change overtime ^#^P<0.05 and ^##^P<0.01. Significant difference between sex *P<0.05 and *P<0.01. | | | | | | | | | | |
|  |  |  |  |  |  |  |  |  |  |  |
