# Supplementary figures and images for: Investigation of the long-term sustainability of changes in appetite after weight loss
Source: Int J Obes (Lond). 2018 Jun 21;42(8):1489–99. doi: 10.1038/s41366-018-0119-9 (PMC6113192; doi:10.1038/s41366-018-0119-9)

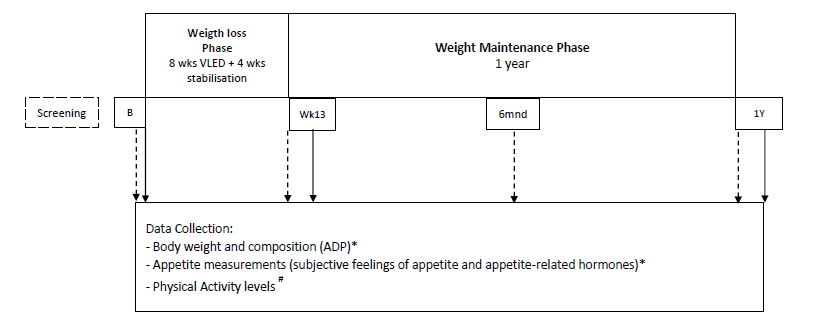

Supplement: Supplementary file 5 — Supplementery Figure I [file 41366_2018_119_MOESM5_ESM.tif]
