# Supplementary material for: Investigation of the long-term sustainability of changes in appetite after weight loss
Source: Int J Obes (Lond). 2018 Jun 21;42(8):1489–99. doi: 10.1038/s41366-018-0119-9 (PMC6113192; doi:10.1038/s41366-018-0119-9)
Supplement: Supplementary file 6 — Supplementery Figure I ledgend [file 41366_2018_119_MOESM6_ESM.docx]

Supplementary figure I. Study diagram. Arrows show data collection time points. VLED: Very low energy diet. Wk: week. B: baseline. Y: year. Mnd: months. ADP: air displacement plethysmography. ADP: Air-displacement plethysmography. ^*^ See solid arrows; ^#^ See dashed arrows.
